# Development and validation of an early diagnostic model to distinguish bacterial infection in patients with acute-on-chronic liver disease (AoCLD) and systemic inflammatory response syndrome (SIRS): a post-hoc analysis of a prospective multicenter cohort

**DOI:** 10.1016/j.jare.2025.10.065

**Published:** 2025-10-28

**Authors:** Hui Zhou, Hai Li, Guohong Deng, Xianbo Wang, Xin Zheng, Jinjun Chen, Zhongji Meng, Yubao Zheng, Yanhang Gao, Zhiping Qian, Feng Liu, Xiaobo Lu, Yu Shi, Jia Shang, Yan Huang, Ruochan Chen

**Affiliations:** a Department of Infectious Diseases, Hunan Key Laboratory of Viral Hepatitis, Xiangya Hospital, Central South University, Changsha, China; b Department of Gastroenterology, School of Medicine, Ren Ji Hospital, Shanghai Jiao Tong University, Shanghai, China; c Key Laboratory of Gastroenterology and Hepatology, Shanghai Institute of Digestive Disease, Chinese Ministry of Health (Shanghai Jiao Tong University), Shanghai, China; d Chinese Chronic Liver Failure (CLIF) Consortium, Shanghai, China; e Department of Infectious Diseases, Southwest Hospital, Third Military Medical University (Army Medical University), Chongqing, China; f Center of Integrative Medicine, Beijing Ditan Hospital, Capital Medical University, Beijing, China; g Department of Infectious Diseases, Institute of Infection and Immunology, Tongji Medical College, Union Hospital, Huazhong University of Science and Technology, Wuhan, China; h Hepatology Unit, Department of Infectious Diseases, Nanfang Hospital, Southern Medical University, Guangzhou, China; i Department of Infectious Diseases, Taihe Hospital, Hubei University of Medicine, Shiyan, China; j Department of Infectious Diseases, The Third Affiliated Hospital, Sun Yat-sen University, Guangzhou, China; k Department of Hepatology, The First Hospital of Jilin University, Changchun, China; l Department of Liver Intensive Care Unit, Shanghai Public Health Clinical Centre, Fudan University, Shanghai, China; m Department of Infectious Diseases and Hepatology, The Second Hospital of Shandong University, Jinan, China; n Infectious Disease Center, The First Affiliated Hospital of Xinjiang Medical University, Ürümqi, China; o State Key Laboratory for Diagnosis and Treatment of Infectious Diseases, Collaborative Innovation Center for Diagnosis and Treatment of Infectious Disease, The First Affiliated Hospital, Zhejiang University School of Medicine, Hangzhou, China; p Department of Infectious Diseases, Henan Provincial People’s Hospital, Zhengzhou, China

**Keywords:** Acute-on-chronic liver disease (AoCLD), Systemic inflammatory response syndrome (SIRS), Infection, Early diagnosis, Risk factors

## Abstract

**Background and objectives:**

Early differentiation between infectious and non-infectious systemic inflammatory response syndrome (SIRS) in patients with acute-on-chronic liver disease (AoCLD) remains challenging. This study aimed to develop and validate an early diagnostic model to accurately identify infection status in AoCLD patients with SIRS, thereby guiding targeted anti-infective therapy and improving clinical management.

**Methods:**

Based on two multicenter prospective cohorts from the Chinese Acute-on-Chronic Liver Failure (CATCH-LIFE) study, 515 AoCLD patients with SIRS were randomly divided into a training cohort (n = 361) and a validation cohort (n = 154). In the training cohort, predictive factors for infection were screened using logistic regression, least absolute shrinkage and selection operator (LASSO) regression, multicollinearity analysis, and stepwise regression. Restricted cubic splines (RCS) were employed to explore nonlinear relationships and interaction effects. A nomogram model was subsequently constructed. The model’s performance was evaluated using receiver operating characteristic (ROC) curves, calibration curves, and decision curve analysis (DCA), with internal validation via bootstrap method.

**Results:**

The final model incorporated five clinical indicators: jaundice, ascites, neutrophil count (N), albumin (ALB), and C-reactive protein (CRP). CRP exhibited a significant nonlinear relationship with infection risk (*P* < 0.001) and was included as a spline term, while an interaction term between ALB and jaundice was incorporated. The model demonstrated excellent discrimination, with area under the ROC curve (AUC) of 0.840 (95 % CI: 0.788–0.888) in the training cohort and 0.865 (95 % CI: 0.803–0.927) in the validation cohort, significantly outperforming single biomarkers. Calibration curves showed high consistency between predicted and observed probabilities. DCA indicated substantial clinical net benefit across threshold probabilities. A clinical decision pathway was developed based on the nomogram’s risk prediction and optimal DCA threshold.

**Conclusion:**

The early diagnostic model developed in this study effectively differentiates between infectious and non-infectious SIRS in AoCLD patients, potentially reducing unnecessary antimicrobial exposure and improving clinical outcomes.

## Highlights

• Developed an early diagnostic model to distinguish infection in AoCLD with SIRS.
• Based on a large, prospective multicenter cohort (CATCH-LIFE, n=515).
• Incorporated five routinely available clinical and laboratory variables.
• Model enables infection risk stratification into low, intermediate, and high groups.
• Supports early identification and targeted intervention to guide antibiotic use.

## Graphical abstract

A practical diagnostic model was developed and validated using the multicenter CATCH-LIFE cohort to distinguish bacterial infection in patients with acute-on-chronic liver disease (AoCLD) and systemic inflammatory response syndrome (SIRS). Based on five routine clinical indicators—jaundice, ascites, neutrophil count, albumin, and CRP—the model demonstrated high accuracy and enables early risk stratification to guide timely antibiotic decisions.

**Figure:**
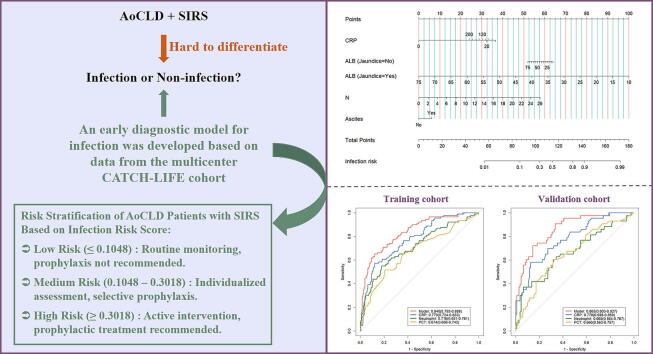

## Introduction

Acute-on-chronic liver disease (AoCLD) is a severe clinical syndrome frequently accompanied by systemic inflammatory response syndrome (SIRS) [1,2]. SIRS can be triggered by infectious or non-infectious factors (such as trauma, pancreatitis, or burns), and is characterized by widespread systemic inflammation [3,4]. Although SIRS is often regarded as a clinical indicator of infection, studies have shown that it is not specific to infection; non-infectious causes can also lead to SIRS [5]. This complexity significantly increases the difficulty of identifying infection status in patients with AoCLD and SIRS.

Bacterial infection is one of the most common complications in patients with AoCLD and is closely associated with increased mortality [6,7]. However, the presence of SIRS does not necessarily indicate infection, yet empirical antibiotic use driven by SIRS misclassification risks antimicrobial resistance and adverse outcomes. Although bacterial culture is the gold standard for diagnosing infection, it is time-consuming and often results in delayed diagnosis and treatment. Misdiagnosis or missed diagnosis may lead to overuse of antibiotics or delayed initiation of targeted anti-infective therapy, thereby worsening disease progression [8,9]. Some studies have attempted to establish infection prediction scoring systems, but most are based on single-center data and have not adequately accounted for the influence of non-infectious factors [10]. This gap impedes timely, precise anti-infective strategies, directly impacting survival and antimicrobial stewardship. Therefore, developing a diagnostic model that accurately distinguishes between infectious and non-infectious SIRS is of significant clinical importance for optimizing personalized treatment strategies in AoCLD patients.

To address this, we leveraged multicenter prospective data from the Chinese Acute-on-Chronic Liver Failure (CATCH-LIFE) study to develop and validate an early diagnostic model for identifying bacterial infection in patients with AoCLD and SIRS. Our goal is to accurately discriminate infectious vs. non-infectious SIRS in AoCLD patients, enabling evidence-based antibiotic initiation to improve survival and curb antimicrobial misuse.

## Methods

### Study design

This study is a retrospective analysis based on two prospective, multicenter cohorts from the CATCH-LIFE study. AoCLD patients with coexisting SIRS who met the inclusion criteria were identified from the prospective cohorts. A retrospective statistical analysis was then conducted based on their clinical characteristics and laboratory findings, with the aim of developing a diagnostic model for the early identification of infection status.

### Study population and data collection

The study population was drawn from two prospective multicenter cohorts within the CATCH-LIFE study. The first cohort included patients enrolled between January 2015 and December 2016 (ClinicalTrials.gov ID: NCT02457637, n = 2600), and the second cohort included patients enrolled between September 2018 and March 2019 (ClinicalTrials.gov ID: NCT03641872, n = 1370), for a total of 3970 patients with AoCLD.

The inclusion and exclusion criteria for AoCLD have been previously described in detail [11,12]. SIRS was diagnosed based on the classical criteria, defined by the presence of two or more of the following four parameters [3,13]: (1) body temperature >38 °C or <36 °C; (2) heart rate >90 beats per minute; (3) respiratory rate >20 breaths per minute or arterial CO_2_ partial pressure <32 mmHg; (4) white blood cell count >12 × 10^9^/L or <4 × 10^9^/L, or the presence of >10 % immature neutrophils.

**Fig. 1:**
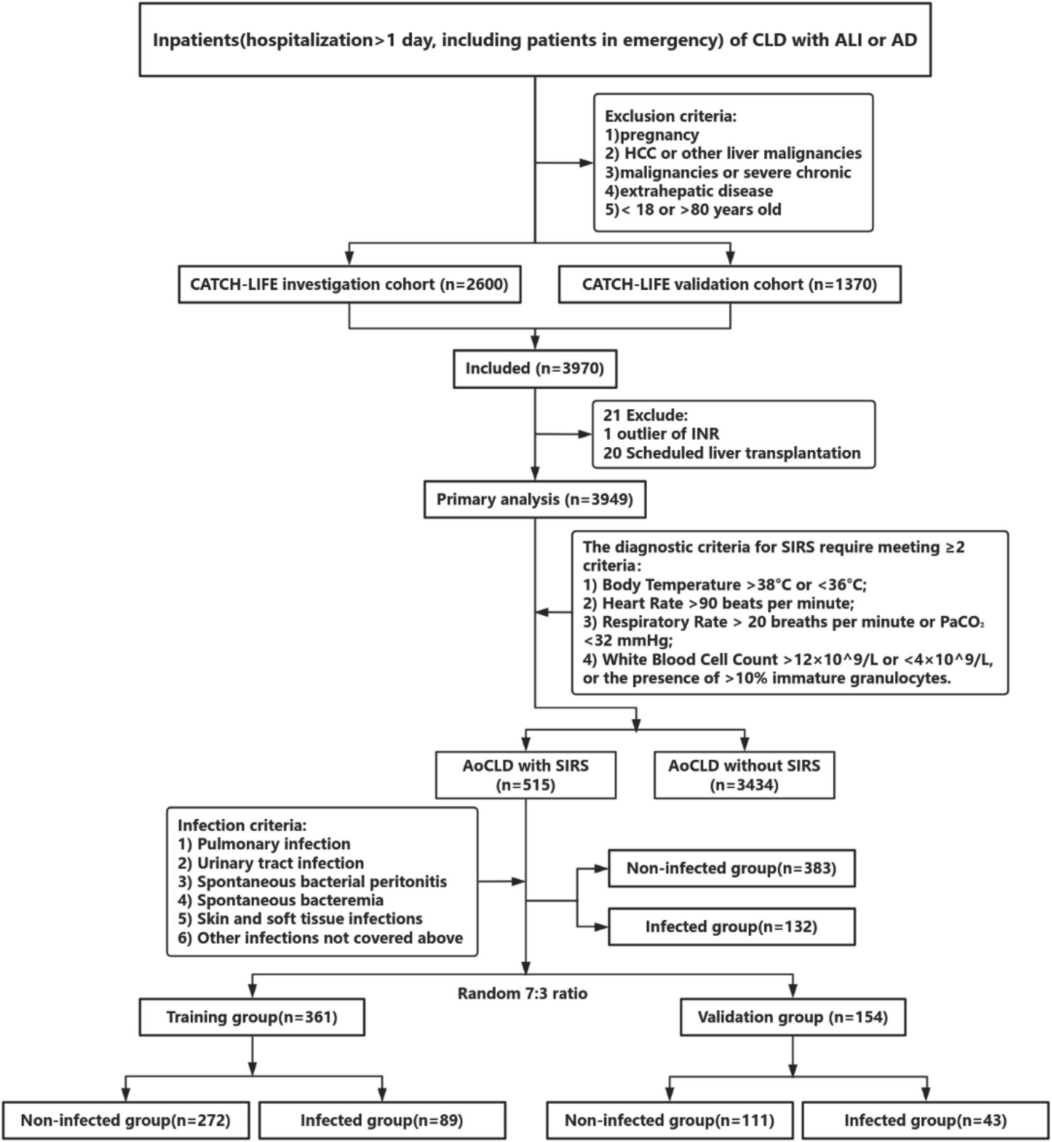
**Flow chart.** CLD, Chronic Liver Disease; ALI, Acute Liver Injury; AD, Acute Decompensation; HCC, Hepatocellular Carcinoma. AoCLD, Acute-on-Chronic Liver Disease; SIRS, Systemic Inflammatory Response Syndrome.

The definition of infection followed established criteria and was detailed in prior studies [14] (Fig. 1). Of the 3970 total AoCLD patients, 3455 were excluded based on the study’s inclusion and exclusion criteria. Ultimately, 515 AoCLD patients with SIRS were included in the final analysis (Fig. 1).

### Missing data handling and sample size estimation

This study first assessed the missing data for each variable in the dataset and visualized the missingness pattern using a flow chart (Fig. S1). To reduce potential bias introduced by missing data, multiple imputation was performed using the “mice” package (version 3.18.0) in R. Five imputed datasets (m = 5) were generated with a maximum of 30 iterations. Predictive Mean Matching (PMM) was used for continuous variables, and logistic regression was used for categorical variables. Convergence was assessed by examining iteration trace plots and the between-chain coefficient of variation. Results showed that 96.6 % of variables achieved excellent convergence (between-chain coefficient of variation < 5 %), and all variables met the convergence criteria (Table S1). No statistically significant differences were observed between the pre- and post-imputation datasets (Table S2).

The sample size was estimated using the “pmsampsize” package in R based on the method proposed by Riley et al. [15]. Input parameters included an outcome event rate of 24.7 % (89/361) in patients with AoCLD complicated by SIRS, an expected C-statistic of 0.840, a Cox–Snell R^2^ value of 0.2644, and 8 candidate predictors. The acceptable difference between the apparent and adjusted R^2^ was set to 0.05, with a margin of error of 0.05 for the intercept estimate. After comprehensive consideration of criteria including the model shrinkage factor and events per predictor (EPP), the minimum required sample size was determined to be 286 (including 71 outcome events, EPP = 8.875). The actual sample size included in this study met this requirement, ensuring sufficient statistical power.

### Ethical approval

This study was conducted in strict accordance with the ethical principles outlined in the Declaration of Helsinki and the Declaration of Istanbul. The study protocol was approved by the Ethics Committee of Renji Hospital, Shanghai Jiao Tong University School of Medicine, China (Approval Nos. 2014-148 K and 2016-142 K). Written informed consent was obtained from all participants prior to enrollment.

### Statistical analysis

A total of 515 patients were included in this study, with 361 randomly assigned to the training cohort for model development and the remaining 154 assigned to the validation cohort for performance evaluation (Fig. 1). There were no significant differences in baseline characteristics between the two cohorts (*P* > 0.05) (Table S3). Continuous variables with a normal distribution were compared using independent sample *t*-tests, while non-normally distributed variables were analyzed using the Mann-Whitney *U* test. Categorical variables were compared using the *χ^2^* test or Fisher’s exact test, as appropriate.

In the training cohort, potential predictive factors were first identified using univariate logistic regression analysis. Subsequently, least absolute shrinkage and selection operator (LASSO) regression, multicollinearity diagnostics, and stepwise regression were employed to further identify independent predictors of infection in patients with AoCLD and SIRS. Multicollinearity was assessed using the criteria of tolerance < 0.1 or variance inflation factor (VIF) > 10[16].

To enhance model performance, restricted cubic splines (RCS) were employed to explore potential nonlinear relationships between continuous variables (including C-reactive protein, albumin, and neutrophil count) and the outcome in the training cohort, while interactions between variables were also tested. Based on the analytical results, the final model form was determined, and a nomogram prediction model was constructed using the “rms” package in R software (v4.3.2).

The model performance was systematically evaluated across multiple dimensions. Discrimination was assessed using the receiver operating characteristic (ROC) curve and the area under the curve (AUC). Calibration was evaluated via calibration curves to examine the agreement between predicted probabilities and observed outcomes. Additionally, decision curve analysis (DCA) was applied to estimate the clinical net benefit across various decision thresholds, thereby validating the model's clinical utility. Optimism correction for model performance metrics was performed using the bootstrap method (with 1,000 resamples) in the training cohort. All statistical tests were two-sided, with a *P*-value < 0.05 considered statistically significant.

Furthermore, individualized infection risk predictions were generated based on the nomogram. The optimal intervention threshold was determined using DCA. By applying an optimized algorithm, continuous predicted probabilities were categorized into three risk tiers—low, medium, and high—with the proportion of medium-risk cases controlled within 30 % [17]. This formed an integrated clinical tool encompassing “risk prediction–risk stratification–decision support.” The workflow involved first calculating an individual’s risk probability, then assigning a risk category, and finally comparing the result with the DCA-derived threshold to generate an intervention recommendation (intervention recommended if ≥ threshold; routine intervention not recommended if < threshold). The stability and clinical utility of this integrated strategy were validated in an independent validation cohort. A cost-effectiveness analysis was also conducted to further evaluate the economic rationality of the strategy.

## Results

### Baseline characteristics of AoCLD patients with SIRS

Among the 515 included AoCLD patients with SIRS, 383 were classified as non-infected and 132 as infected. The infected group had a higher mean age compared to the non-infected group (51.9 vs 47.3 years, *P* < 0.001). The proportion of cirrhosis was significantly greater in the infected group than in the non-infected group [113 (85.6 %) vs 251 (65.5 %), *P* < 0.001]. Regarding acute decompensation events, the infected group showed higher rates of jaundice [95 (72.0 %) vs 164 (42.8 %), *P* < 0.001] and ascites [92 (69.7 %) vs 156 (40.7 %), *P* < 0.001] compared to the non-infected group. Additionally, neutrophil count (N), C-reactive protein (CRP), and procalcitonin (PCT) levels were significantly elevated in the infected group (*P* < 0.001), whereas albumin (ALB) levels were significantly lower than those in the non-infected group (*P* < 0.001). Coagulation function indicators, including prothrombin time (PT) and international normalized ratio (INR), were also significantly higher in the infected group compared to the non-infected group (*P* < 0.001) (Table 1).

**Table 1:** Comparison of baseline characteristics between infected and uninfected patients with AoCLD combined with SIRS.

**Characteristics**	**Non-infected group**	**Infected group**	***P* value**
	**(n = 383)**	**(n = 132)**	
Demographic data			
Age	47.3 ± 11.8	51.9 ± 11.9	<0.001***
Male	294 (76.8 %)	91 (68.9 %)	0.095
Aetiology			
HBV	284 (74.2 %)	87 (65.9 %)	0.088
HCV	13 (3.39 %)	2 (1.52 %)	0.375
HEV	7 (1.83 %)	4 (3.03 %)	0.484
Alcohol	62 (16.2 %)	31 (23.5 %)	0.080
Autoimmune	36 (9.40 %)	21 (15.9 %)	0.058
NAFLD	17 (4.44 %)	3 (2.27 %)	0.396
Schistosomiasis	1 (0.26 %)	2 (1.52 %)	0.163
Cryptogenic	22 (5.74 %)	7 (5.30 %)	1.000
DILI	3 (0.78 %)	0 (0.00 %)	0.573
Cirrhosis			
Yes	251 (65.5 %)	113 (85.6 %)	<0.001***
AD			
HE			0.053
non-HE	360 (94.0 %)	117 (88.6 %)	
Grade 1–2	19 (4.96 %)	10 (7.58 %)	
Grade 3–4	4 (1.04 %)	5 (3.79 %)	
Jaundice	164 (42.8 %)	95 (72.0 %)	<0.001***
Ascites	156 (40.7 %)	92 (69.7 %)	<0.001***
GI bleeding	57 (14.9 %)	12 (9.09 %)	0.124
ACLF			
Yes	49 (12.8 %)	47 (35.6 %)	<0.001***
Blood routine			
WBC (×10^9^/L)	4.61 [3.47;6.56]	6.80 [4.46;9.66]	<0.001***
N (×10^9^/L)	2.79 [1.77;4.23]	4.88 [2.66;7.89]	<0.001***
M (×10^9^/L)	0.39 [0.26;0.60]	0.50 [0.27;0.79]	0.005**
N (%)	61.5 [53.0;71.0]	73.7 [63.2;80.8]	<0.001***
M (%)	8.60 [6.62;11.2]	7.80 [5.70;10.7]	0.053
PLT (×10^9^/L)	98.0 [59.5;152]	85.0 [50.0;131]	0.058
HGB (g/L)	120 [100;137]	110 [89.0;127]	0.002**
NLR	2.19 [1.48;3.77]	4.38 [2.40;6.89]	<0.001***
Liver function			
ALB (g/L)	33.2 [29.0;37.8]	29.2 [25.7;33.5]	<0.001***
TB (mg/dL)	3.60 [1.71;12.9]	13.1 [3.95;23.6]	<0.001***
ALT (U/L)	113 [33.0;576]	76.0 [35.4;225]	0.037*
AST (U/L)	146 [48.5;336]	117 [53.1;237]	0.373
AST/ALT	1.07 [0.63;1.70]	1.40 [0.93;2.04]	<0.001***
AKP (U/L)	126 [93.7;172]	142 [98.0;184]	0.145
γ-GT (U/L)	81.0 [40.2;154]	73.6 [40.8;126]	0.311
Inflammation indication			
CRP (mg/L)	6.42 [2.84;11.7]	18.8 [8.70;32.2]	<0.001***
PCT (ng/mL)	0.19 [0.12;0.47]	0.50 [0.19;0.93]	<0.001***
Coagulation function			
PT(s)	15.8 [13.1;19.9]	19.3 [15.3;25.2]	<0.001***
INR	1.35 [1.17;1.67]	1.70 [1.34;2.34]	<0.001***
Kidney function			
Cr (mg/dL)	0.74 [0.63;0.87]	0.79 [0.60;1.09]	0.043*
BUN (mmol/L)	4.30 [3.29;5.90]	5.05 [3.40;9.00]	0.003**
BUN/Cr	5.88 [4.57;8.14]	6.65 [4.73;9.51]	0.061
eGFR (mL/min)	108 [96.8;117]	97.2 [76.5;113]	<0.001***
Electrolyte			
K^+^ (mmol/L)	3.90 [3.50;4.24]	3.80 [3.39;4.20]	0.191
Na^+^ (mmol/L)	139 [136;141]	136 [132;139]	<0.001***
Vital signs			
OI (mmHg)	476 [467;476]	476 [467;476]	0.047*

Note: * represents *P* < 0.05, ** represents *P* < 0.01, and *** represents *P* < 0.001. ACLF, acute-on-chronic liver failure; ALT, alanine aminotransferase; AST, aspartate aminotransferase; AST/ALT, aspartate to alanine aminotransferase ratio; ALB, albumin; AKP, alkaline phosphatase; BUN, blood urea nitrogen; CRP, C-reactive protein; Cr, creatinine; DILI, drug-induced liver impairment; eGFR. glomerular filtration rate; GI bleeding, gastrointestinal bleeding; HBV, hepatitis B virus; HCV, hepatitis C virus; HEV, hepatitis E virus; HE, hepatic encephalopathy; HGB, hemoglobin; INR, International Normalized Ratio; K^+^, blood potassium; M, monocyte count; M (%), monocyte percentage; N, neutrophil count; N (%), percent neutrophils; NLR, neutrophil-to-lymphocyte ratio; NAFLD, nonalcoholic fatty liver disease; Na^+^, blood sodium; OI, oxygenation index; PLT, platelet; PCT, procalcitonin; PT, plasminogen time; TB, total bilirubin; WBC, white blood cell.

### Variable selection

#### Preliminary variable screening based on univariate logistic regression

Univariate logistic regression analysis showed that age, alcohol intake, autoimmune liver disease, cirrhosis, jaundice, ascites, acute-on-chronic liver failure (ACLF), white blood cell count (WBC), N, neutrophil percentage (N%), monocyte count (M), hemoglobin (HGB), ALB, total bilirubin (TB), aspartate aminotransferase to alanine aminotransferase ratio (AST/ALT), gamma-glutamyl transferase (γ-GT), CRP, PCT, PT, INR, creatinine (Cr), blood urea nitrogen (BUN), estimated glomerular filtration rate (eGFR), and blood sodium (Na^+^) levels were all significantly associated with infection occurrence in AoCLD patients with SIRS (*P* < 0.05) (Table 2).

**Table 2:** Infection-related risk factors in a training cohort of patients with AoCLD combined with SIRS were analyzed using univariate logistic regression analysis.

**Characteristics**	**Univariate logistic regression**		
	**OR**	**95 %CI**	***P* value**
Demographic data			
Age	1.03	1.01–1.05	0.014*
Gender	0.85	0.50–1.48	0.563
Aetiology			
HBV	0.65	0.39–1.08	0.090
HCV	0.30	0.02–1.59	0.251
HEV	1.23	0.17–5.81	0.808
Alcohol	2.09	1.14–3.75	0.015*
Autoimmune	2.14	1.09–4.12	0.024*
NAFLD	0.50	0.08–1.87	0.368
Schistosomiasis	6.23	0.59–135.02	0.137
Cryptogenic	0.84	0.27–2.18	0.738
DILI	5.32e + 08	0.00-NA	0.999
Cirrhosis	2.61	1.43–5.04	0.003**
AD			
HE Grade 1–2	1.46	0.54–3.60	0.423
HE Grade 3–4	4.22	0.91–21.78	0.063
Jaundice	3.8	2.26–6.58	<0.001***
Ascites	2.33	1.43–3.87	0.001**
GI bleeding	0.56	0.23–1.18	0.150
ACLF	3.42	1.98–5.91	<0.001***
Blood routine			
WBC (×10^9^/L)	1.23	1.15–1.32	<0.001***
N (×10^9^/L)	1.31	1.20–1.44	<0.001***
N (%)	2.96	1.60–5.63	<0.001***
M (×10^9^/L)	1.06	1.04–1.09	0.001**
M (%)	0.96	0.89–1.02	0.194
PLT (×10^9^/L)	1.00	1.00–1.00	0.646
HGB (g/L)	0.99	0.98–1.00	0.041*
NLR	1.04	1.00–1.09	0.142
Liver function			
ALB (g/L)	0.90	0.87–0.94	<0.001***
TB (mg/dL)	1.05	1.03–1.08	<0.001***
ALT (U/L)	1.00	1.00–1.00	0.477
AST (U/L)	1.00	1.00–1.00	0.787
AST/ALT	1.43	1.16–1.79	0.001**
AKP (U/L)	1.00	1.00–1.00	0.590
γ-GT (U/L)	1.00	1.00–1.00	<0.001***
Inflammation indication			
CRP (mg/L)	1.04	1.02–1.05	<0.001***
PCT (ng/mL)	1.62	1.27–2.19	<0.001***
Coagulation function			
PT (s)	1.04	1.02–1.07	0.001**
INR	1.55	1.17–2.15	0.005**
Kidney function			
Cr (mg/dL)	2.10	1.34–3.53	0.003**
BUN (mmol/L)	1.12	1.06–1.19	<0.001***
BUN/Cr	1.00	0.99–1.02	0.478
eGFR (mL/min)	0.99	0.98–1.00	0.021*
Electrolyte			
K^+^ (mmol/L)	1.24	0.85–1.84	0.275
Na^+^ (mmol/L)	0.90	0.85–0.94	<0.001***
Vital signs			
OI (mmHg)	1.00	0.99–1.00	0.348

Note: * represents *P* < 0.05, ** represents *P* < 0.01, and *** represents *P* < 0.001. ACLF, acute-on-chronic liver failure; ALT, alanine aminotransferase; AST, aspartate aminotransferase; AST/ALT, aspartate to alanine aminotransferase ratio; ALB, albumin; AKP, alkaline phosphatase; BUN, blood urea nitrogen; CRP, C-reactive protein; Cr, creatinine; DILI, drug-induced liver impairment; eGFR. glomerular filtration rate; GI bleeding, gastrointestinal bleeding; HBV, hepatitis B virus; HCV, hepatitis C virus; HEV, hepatitis E virus; HE, hepatic encephalopathy; HGB, hemoglobin; INR, International Normalized Ratio; K^+^, blood potassium; M, monocyte count; M (%), monocyte percentage; N, neutrophil count; N (%), percent neutrophils; NLR, neutrophil-to-lymphocyte ratio; NAFLD, nonalcoholic fatty liver disease; Na^+^, blood sodium; OI, oxygenation index; PLT, platelet; PCT, procalcitonin; PT, plasminogen time; TB, total bilirubin; WBC, white blood cell.

#### LASSO regression analysis

Variables with statistically significant differences (*P* < 0.05) in the univariate logistic regression analysis were further included in the LASSO regression analysis. The results showed that when the λ value was 0.0278, the model demonstrated optimal variable selection performance (Figs. S2A and S2B). The selected variables included age, autoimmune liver disease, cirrhosis, jaundice, ascites, ACLF, N, N%, ALB, CRP, PT, BUN, and Na^+^, all with nonzero coefficients (Table 3).

**Table 3:** Variables with non-zero regression coefficients selected by LASSO regression analysis.

**Charactor**	**Active**
Age	0.001
Autoimmune	0.510
Cirrhosis	0.048
Jaundice	0.625
Ascites	0.054
ACLF	0.179
N (×10^9^/L)	0.117
N (%)	0.006
ALB (g/L)	−0.052
CRP (mg/L)	0.009
PT (s)	0.0002
BUN (mmol/L)	0.023
Na^+^ (mmol/L)	−0.009
Constant	−0.096

Note: ACLF, acute-on-chronic liver failure; ALB, albumin; BUN, blood urea nitrogen; CRP, C-reactive protein; N, neutrophil count; N (%), percent neutrophils; Na^+^, blood sodium; PT, plasminogen time.

#### Multicollinearity assessment

Multicollinearity was assessed for the feature variables selected through the LASSO regression analysis. The results showed that all variables had tolerance values greater than 0.1 and VIFs less than 10, indicating no significant multicollinearity among the variables (Table 4).

**Table 4:** Check for multicollinearity.

**Variables**	**Tolerance**	**VIF**
Age	0.790	1.266
Autoimmune	0.941	1.063
Cirrhosis	0.637	1.569
Jaundice	0.734	1.362
Ascites	0.705	1.419
ACLF	0.652	1.533
N (×10^9^/L)	0.513	1.951
N (%)	0.549	1.822
ALB (g/L)	0.735	1.360
CRP (mg/L)	0.779	1.284
PT (s)	0.691	1.446
BUN (mmol/L)	0.751	1.332
Na^+^ (mmol/L)	0.827	1.209

Note: ACLF, acute-on-chronic liver failure; ALB, albumin; BUN, blood urea nitrogen; CRP, C-reactive protein; N, neutrophil count; N (%), percent neutrophils; Na^+^, blood sodium; PT, plasminogen time.

#### Stepwise regression analysis

Based on the results of the LASSO regression analysis, a stepwise logistic regression was performed on the selected variables. The results showed that jaundice (OR = 2.77, 95 % CI = 1.52–5.19, *P* < 0.001), ascites (OR = 1.45, 95 % CI = 0.80–2.63, *P* = 0.219), N (OR = 1.20, 95 % CI = 1.09–1.33, *P* < 0.001), ALB (OR = 0.92, 95 % CI = 0.88–0.97, *P* < 0.001), and CRP (OR = 1.02, 95 % CI = 1.00–1.03, *P* = 0.015) were independent risk factors for infection in AoCLD patients with SIRS (Table 5).

**Table 5:** Stepwise logistic regression.

**Variables**	**Stepwise logistic regression**		
	**OR**	**95 %CI**	***P***
Jaundice	2.77	1.52–5.19	<0.001***
Ascites	1.45	0.80–2.63	0.219
N (×10^9^/L)	1.20	1.09–1.33	<0.001***
ALB (g/L)	0.92	0.88–0.97	<0.001***
CRP (mg/L)	1.02	1.00–1.03	0.015*
Constant	2.459		0.261

Note: ALB, albumin; CRP, C-reactive protein; N, neutrophil count.

Although ascites did not reach statistical significance in the stepwise regression (*P* = 0.219), its inclusion improved the AUC of the training cohort from 0.834 to 0.840 (Fig. S3). Furthermore, studies have shown that bacterial infections can trigger ascites formation in cirrhotic patients [18], while the presence of ascites may in turn significantly increase the risk of infection by promoting bacterial translocation [19]. Given this bidirectional relationship and its important clinical implications, ascites was retained as a key variable in the final diagnostic model.

### Model construction

**Fig. 2:**
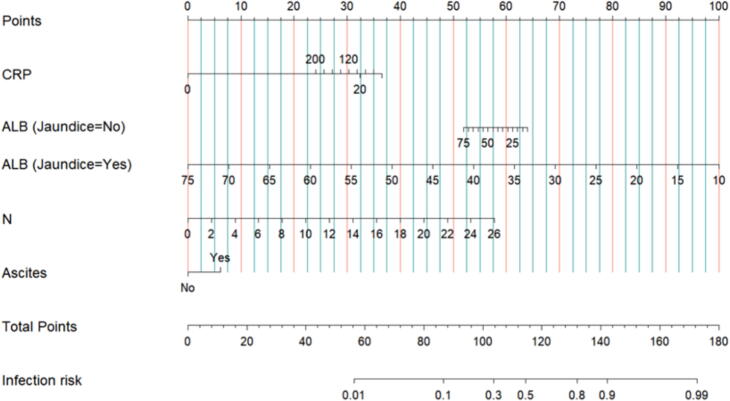
**Nomogram for early diagnosis of infection in patients with AoCLD combined with SIRS.** ALB, Albumin; CRP, C-reactive Protein; N, Neutrophils.

The key variables included in this study for model construction were jaundice, ascites, ALB, CRP, and N. To optimize model performance, restricted cubic splines (RCS) were used to analyze potential nonlinear relationships between ALB, CRP, and N and the risk of infection. Results revealed a significant nonlinear association between CRP and infection risk (nonlinearity test *P* < 0.001); therefore, CRP was incorporated into the model as a spline term. In contrast, ALB and N were included as linear terms (Table S4). Several pre-specified interaction effects were tested, among which a significant interaction between ALB and jaundice status was identified (interaction term *P* = 0.048) (Table S5). Based on these findings, a final simplified model was developed, incorporating the CRP spline term and the ALB-jaundice interaction term. Regression coefficients and associated statistics are presented in Table S6. Furthermore, the simplified model was compared against a full model containing all variables using the Akaike Information Criterion (AIC) and Bayesian Information Criterion (BIC). The simplified model demonstrated superior performance on both metrics (Table S7), indicating better fit and greater parsimony. Based on these results, a nomogram model was constructed for early discrimination of infected versus non-infected patients with AoCLD and SIRS (Fig. 2). An online calculation tool (https://cld-infection.shinyapps.io/AoCLD_SIRS_DynNomapp/) was also developed to facilitate clinical use.

### Model evaluation and validation

**Fig. 3:**
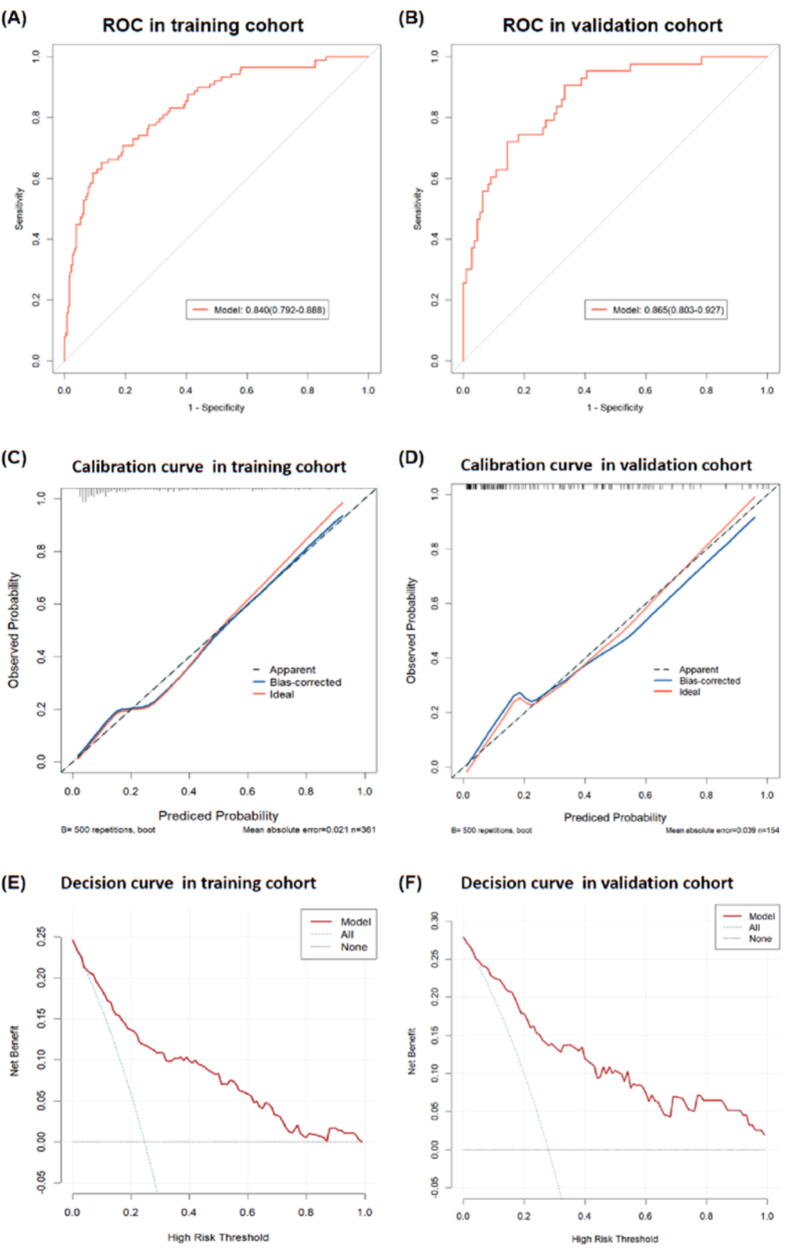
**Model Evaluation and Validation.** (A, B) ROC curves of the model in the training and validation cohorts. (C, D) Calibration curves of the model in the training and validation cohorts. The x-axis represents the predicted probability, and the y-axis represents the observed probability. An ideal prediction model would yield a calibration curve along the 45° diagonal line (ideal line), indicating perfect agreement between predicted and actual probabilities. The “Apparent” line reflects the model’s performance in the training set, while the “Bias-corrected” line represents the performance after repeated bootstrapping. If both lines closely follow the ideal line without significant deviation, the model is considered well-calibrated. (E, F) Decision curve analyses for the training and validation cohorts. The x-axis represents the threshold probability, and the y-axis represents the net benefit. The dashed line denotes the “treat all” strategy, the dotted line denotes the “treat none” strategy, and the solid line represents the model.

The diagnostic performance of the model was evaluated in both the training and validation cohorts by plotting ROC curves, with the training cohort curve further adjusted using 1,000 bootstrap resamples. The results showed that the AUCs with 95 % confidence intervals (CI) were 0.840 (95 % CI: 0.788–0.888) in the training cohort and 0.865 (95 % CI: 0.803–0.927) in the validation cohort, indicating high diagnostic accuracy (Fig. 3, Fig. 3). In the training cohort, the original AUC was 0.840 and the original calibration slope was 1. After bootstrap optimism correction, the optimism-corrected AUC remained 0.840, the Brier score was 0.127, and the corrected calibration slope was 1.097. These corrected metrics suggest that the model exhibits good discrimination and calibration, with a low risk of overfitting.

Calibration curve analysis demonstrated excellent agreement between predicted and observed risks in both the training and validation cohorts (Fig. 3, Fig. 3). Decision curve analysis (DCA) further indicated that the model provided positive net clinical benefits across a wide range of threshold probabilities, with an optimal intervention threshold identified at 1 % (Fig. 3, Fig. 3). This relatively low threshold may reflect clinical rationality in a context where the consequences of infection are severe and the risks of preventive intervention are relatively low, supporting a more liberal intervention strategy. In summary, the infection risk prediction model developed in this study demonstrates strong performance in discrimination, calibration, and clinical utility, showing considerable potential for broad application. It can provide individualized support for early infection identification and intervention decision-making in patients with AoCLD complicated by SIRS.

### Comparison of diagnostic performance with other indicators

**Fig. 4:**
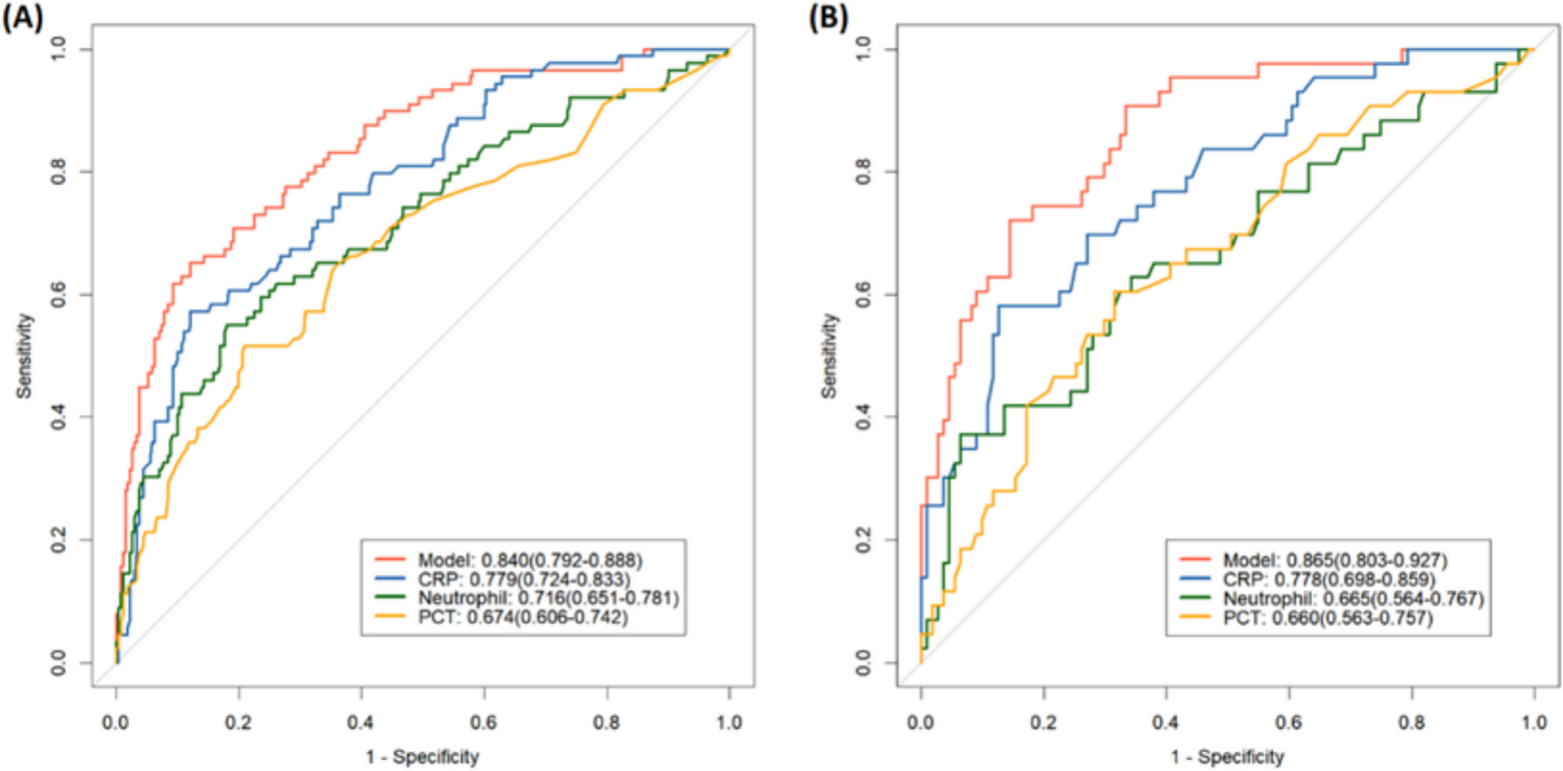
**Comparison of ROC curves for the model, CRP, N, and PCT.** (A) Training cohort; (B) Validation cohort.

To evaluate the diagnostic performance of the model, we compared its AUC with those of other commonly used indicators. In the training cohort, the AUCs (95 % CI) for the model, CRP, N, and PCT were 0.840 (0.792–0.888), 0.779 (0.724–0.833), 0.716 (0.651–0.767), and 0.660 (0.563–0.757), respectively (Fig. 4A). In the validation cohort, the AUCs (95 % CI) for the model, CRP, N, and PCT were 0.865 (0.803–0.927), 0.778 (0.698–0.859), 0.665 (0.564–0.767), and 0.660 (0.563–0.757), respectively (Fig. 4B). The models in both the training and validation cohorts demonstrated significantly higher AUCs than CRP, N, and PCT (*P* < 0.05).

### A strategy for rapid identification and management of infection in patients with AoCLD complicated by SIRS

Based on the infection risk probabilities derived from the training cohort nomogram and the optimal threshold determined by DCA, the following clinical decision pathway was established: patients in the low-risk category (≤0.1048) are advised to undergo routine monitoring without routine prophylactic treatment; those in the medium-risk category (0.1048–0.3018) are recommended for individualized assessment, with selective prophylaxis considered; and high-risk patients (≥0.3018) are recommended for proactive preventive interventions (Table 6). In the training cohort, the proportions of low-, medium-, and high-risk patients were 42.4 %, 24.9 %, and 32.7 %, respectively, indicating that the stratification system possesses good discriminative ability and clinical feasibility (Table 6). The corresponding proportions in the validation cohort were 38.3 %, 24.0 %, and 37.7 %, further demonstrating its stability and generalizability across different populations (Table 6).

**Table 6:** Risk Stratification and Decision Support Based on the Nomogram and Decision Curve Analysis.

**Risk Category**	**Probability Range**	**Training Cohort (n = 361)**			**Validation Cohort (n = 154)**		**Clinical Management**
		**Proportion**	**Infection Rates**		**Proportion**	**Infection Rates**	
Low Risk	≤0.1048	42.4 %	5.9 %		38.3 %	3.4 %	Routine monitoring, prophylaxis not recommended
Medium Risk	0.1048 ∼ 0.3018	24.9 %	18.9 %		24.0 %	25.0 %	Individualized assessment, selective prophylaxis
High Risk	≥0.3018	32.7 %	53.4 %		37.7 %	55.2 %	Active intervention, prophylactic treatment recommended

Note: The optimal decision threshold determined by DCA was 1.0%. The predicted probabilities for all risk strata exceeded this threshold, supporting prophylactic intervention across all risk categories.

To further evaluate the economic rationality of this strategy, a cost-benefit analysis was conducted. Although precise cost data were difficult to obtain, an analysis from a clinical benefit perspective revealed that at the optimal threshold of 1 %, the number needed to treat (NNT) to prevent one infection case was 3.6. This value falls within the generally accepted range in the field of infection prevention.

To validate the clinical application value of the model, clinical data from one non-infected patient and one infected patient with AoCLD and SIRS were selected for analysis using the online calculation tool (https://cld-infection.shinyapps.io/AoCLD_SIRS_DynNomapp/). The results showed that the predicted risk for the non-infected patient was 0.02081, classifying them as low-risk and suggesting routine monitoring without prophylactic intervention. In contrast, the infected patient had a predicted risk of 0.98700, placing them in the high-risk category and warranting a recommendation for active preventive intervention (Fig. S4). This outcome confirms the model's strong clinical discriminative capacity.

## Discussion

This study addresses the critical need to accurately distinguish between infectious and non-infectious SIRS in patients with acute-on-chronic liver disease (AoCLD)—a distinction complicated by the dual role of SIRS, which is both a consequence of infection and a catalyst for secondary infections through immune dysregulation and gut microbial translocation[[20], [21], [22]]. It is particularly important to note that non-infected SIRS controls themselves may harbor subclinical inflammation, driven by factors such as intestinal barrier disruption[23], which presents an inherent challenge to the specific discrimination of biomarkers. Failure to differentiate these states fuels antibiotic overuse, accelerates antimicrobial resistance, and delays targeted therapy, directly impacting mortality. Leveraging multicenter prospective data, we developed the first nomogram model integrating five readily available variables (jaundice, ascites, ALB, CRP, N) to enable early, evidence-based infection diagnosis.

The model’s variables—all of which are readily accessible in clinical practice—reflect synergistic pathways linking SIRS to infection risk. Jaundice and ascites are hallmark features of acute decompensation in AoCLD [24], and often serve as early clinical indicators of infection susceptibility. Studies have shown that jaundice may lead to gut microbiota dysbiosis due to reduced bile acid secretion, promoting bacterial translocation [25,26]. Meanwhile, the dysfunction of innate immune cells such as neutrophils and natural killer cells [27,28] contributes to immune paralysis, markedly increasing the risk of infection. Although ascites did not reach statistical significance in the stepwise regression analysis (*P* = 0.219), we retained it in the final model based on a compelling clinical and pathophysiological rationale. From a clinical perspective, ascites is not only a hallmark of decompensated cirrhosis but also a crucial indicator for predicting infection risk, particularly spontaneous bacterial peritonitis (SBP) [29,30]. Mechanistically, the presence of ascites is closely linked to intestinal barrier dysfunction, facilitating bacterial translocation, which is a key initiating event for SBP [31]. Furthermore, the ascitic microenvironment in cirrhosis is inherently immunosuppressive, characterized by reduced complement levels, impaired macrophage phagocytosis, and increased immune cell apoptosis, collectively leading to diminished antimicrobial clearance capacity and creating a condition ripe for secondary infection [32,33]. Therefore, including this readily assessable clinical sign significantly enhances the model's clinical utility and operational practicality.

Hypoalbuminemia is not only a contributing factor to ascites formation [34] but also a key marker of disease severity in advanced liver disease, independently predicting adverse outcomes [35]. Albumin exerts immunomodulatory effects by binding endotoxins and inflammatory mediators; hypoalbuminemia compromises this protective mechanism, thereby increasing infection risk [36,37]. Notably, this study further identified a significant interaction between albumin and jaundice (*P* < 0.05). Patients with coexisting hypoalbuminemia and jaundice exhibited a significantly higher risk of infection compared to those with only one abnormality. This interaction reflects a synergistic pathological process involving dual decompensation of hepatic synthetic and excretory functions: hypoalbuminemia impairs immunoregulation and bilirubin conjugation capacity, while bilirubin accumulation further exacerbates immunosuppression, collectively increasing susceptibility to infection.

CRP, PCT, and neutrophil count are classic infection biomarkers, and their elevation typically suggests bacterial infection [38,39]. However, in AoCLD patients with SIRS, the diagnostic performance of these markers is affected by underlying immune dysfunction and systemic inflammatory responses. This study identified a significant nonlinear relationship between CRP and infection risk (*P* < 0.001). The predictive value of CRP increased steadily within low to moderate concentration ranges, while beyond a certain threshold, the infection risk exhibited exponential growth. This finding explains why CRP outperforms PCT and neutrophil count in distinguishing between infected and non-infected states, yet still lacks sufficient specificity when used alone—although CRP is markedly elevated in infection [40], it can also rise in non-infectious SIRS due to sterile inflammation [41]. PCT, while highly specific, may have lower discriminative ability than CRP in early differentiation between infectious and non-infectious SIRS [41]; neutrophil count, although sensitive in early infection, lacks specificity and is inadequate as a standalone diagnostic marker. In contrast, by incorporating the nonlinear effect of CRP into the model using RCS, we not only more accurately captured the dynamic predictive capacity of its concentrations but also effectively integrated it with liver disease-specific indicators such as jaundice and ascites. This multivariable integration strategy successfully overcomes the limitations of individual biomarkers in complex clinical settings, contributing to the model's superior discriminative performance and clinical applicability in patients with AoCLD complicated by SIRS.

Based on the infection risk probabilities derived from the training cohort nomogram and the optimal threshold identified by DCA, this study established a structured clinical decision pathway designed to enable early risk stratification and precise intervention for patients with AoCLD complicated by SIRS. This pathway categorizes patients into three tiers—low, medium, and high risk—with corresponding recommendations for routine monitoring, individualized assessment with consideration of selective prophylaxis, and active targeted preventive measures, respectively. To facilitate the translation of this model into real-world clinical practice, we developed an interactive web-based calculator (https://cld-infection.shinyapps.io/AoCLD_SIRS_DynNomapp/). By entering the relevant parameters, clinicians can obtain real-time infection risk probabilities and corresponding stratified management recommendations. This tool not only enhances the accessibility and practicality of the model but also helps standardize antibiotic use in AoCLD patients, thereby improving the overall efficiency of healthcare resource allocation.

This study has several strengths. First, it is based on a large sample size and multicenter prospective cohorts with high-quality data, enhancing the model’s generalizability and external applicability. The model performed well in both the training and validation cohorts, indicating strong robustness. Second, the established structured risk-stratification and management pathway enables clinicians to rapidly identify patients at different risk levels during actual clinical practice, thereby improving the efficiency and accuracy of bedside decision-making.

Nevertheless, this study has several limitations. First, although the data were derived from a prospective multicenter cohort, the analytical process was inherently based on retrospective modeling of existing data. The observational study design limits direct causal inference [42], and residual selection bias or information bias may remain uncontrolled. Second, to balance clinical feasibility and cost-effectiveness, the variables selected for the model were limited to routine clinical and laboratory parameters. Future studies could incorporate novel biomarkers such as cytokine profiles or microbiome markers [43,44] to further improve the model's discriminative accuracy. Finally, both the training and validation cohorts were derived from the same study population. The generalizability of the model has not been fully established, and external validation in larger, more geographically diverse cohorts of patients with AoCLD and SIRS of varying etiologies is necessary to comprehensively evaluate its universality and robustness.

## Conclusion

The nomogram model developed in this study provides an effective tool for clinicians to identify the infectious status of AoCLD patients with SIRS at an early stage, significantly improving the accuracy and efficiency of infection diagnosis. It contributes to the rational use of antibiotics, optimization of treatment strategies, and improved patient outcomes. Moreover, the proposed risk stratification strategy offers strong support for clinical decision-making in complex cases.

## Ethics approval

This study was carried out strictly following the ethical requirements specified in the Declaration of Helsinki and the Declaration of Istanbul. The study protocol has been reviewed and approved by the Ethics Committee of Renji Hospital, Shanghai Jiao Tong University School of Medicine, China, with two approval numbers: 2014-148K and 2016-142K. Before participant recruitment, written informed consent was collected from every enrolled subject.

## Financial support and sponsorship

This work was supported by the National Natural Science Foundation of China (Grant numbers 82070613 and 82370638 to RC), the Hunan Science and Technology Innovation Program (Grant number 2022RC1212 to RC), and the Hunan Provincial Natural Science Foundation (Grant number 2023JJ10095 to RC).

## Declaration of competing interest

The authors declare that they have no known competing financial interests or personal relationships that could have appeared to influence the work reported in this paper.
