# Supplementary material for: Development and validation of an early diagnostic model to distinguish bacterial infection in patients with acute-on-chronic liver disease (AoCLD) and systemic inflammatory response syndrome (SIRS): a post-hoc analysis of a prospective multicenter cohort
Source: J Adv Res. 2025 Oct 28;85:469–79. doi: 10.1016/j.jare.2025.10.065 (PMC13316489; doi:10.1016/j.jare.2025.10.065)
Supplement: Supplementary Data 1 [file mmc1.docx]

**Supplementary materials**

**Development and Validation of an Early Diagnostic Model to Distinguish Bacterial Infection in Patients with Acute-on-Chronic Liver Disease (AoCLD) and Systemic Inflammatory Response Syndrome (SIRS): A Post-hoc Analysis of a Prospective Multicenter Cohort**

**Tables：**

**Table S1. Technical Details of Multiple Imputation (MICE) and Convergence Diagnosis Results.**

| **Item** | **Details** |  |
| --- | --- | --- |
| **Basic Data Information** | |  |
| Sample size | 515 cases |  |
| Number of variables | 44 |  |
| Proportion of missing values | 3% |  |
| **Imputation Parameter Settings** | |  |
| Imputation method | Multiple Imputation by Chained Equations (MICE) |  |
| Software version | mice 3.18.0 |  |
| Number of imputed datasets | 5 |  |
| Random seed | 6666 |  |
| Convergence criterion | Inter-chain coefficient of variation (CV) < 5% |  |
| **Main Imputation Methods** |  |  |
| Continuous variables | Predictive Mean Matching (PMM) |  |
| Binary variables | Logistic regression (logreg) |  |
| **Convergence Diagnosis Results** | |  |
| Total number of variables | 29 |  |
| Excellent convergence (CV < 5%) | 28 (96.6%) |  |
| Acceptable convergence (CV 5-10%) | 1 (3.4%) |  |
| Poor convergence (CV ≥ 10%) | 0 (0.0%) |  |
| Overall convergence status | Excellent |  |

**Table S2. Comparison of distribution characteristics of variables before and after missing data imputation in patients with AoCLD complicated with SIRS.**

| **variables** | **Dataset 1** | **Dataset 2** | ***P* value** |
| --- | --- | --- | --- |
|  |  |  |  |
| DILI | 3 (0.5%) | 3 (0.5%) | 0.998 |
| ACLF | 91 (17.6%) | 96 (18.6%) | 0.258 |
| WBC (×10^9^/L) | 5.9517±3.7542 | 5.9517±3.7542 | 0.949 |
| N (%) | 64.4489±13.97534 | 64.1812±14.29388 | 0.947 |
| M (%) | 8.8907±3.83455 | 8.8293±3.84762 | 0.937 |
| PLT (×10^9^/L) | 113.3577±98.86512 | 113.1852±98.71259 | 0.995 |
| HGB (g/L) | 114.8733±28.46848 | 114.9515±28.44176 | 0.978 |
| NLR | 4.057±6.48466 | 4.0283±6.44251 | 0.996 |
| ALB (g/L) | 32.3221±6.78154 | 32.3944±6.81673 | 0.984 |
| TB (mg/dL) | 9.896±10.27413 | 9.8809±10.26986 | 0.98 |
| ALT (U/L) | 366.3386±612.49795 | 367.1573±612.18388 | 0.989 |
| AST (U/L) | 285.985±443.00373 | 285.985±443.00373 | 1 |
| AST/ALT | 1.3921±1.06978 | 1.3904±1.06948 | 0.983 |
| AKP (U/L) | 148.3723±95.22089 | 148.2563±94.45616 | 0.912 |
| γ-GT (U/L) | 123.7686±168.25768 | 122.9323±165.85394 | 0.867 |
| CRP (mg/L) | 14.9629±21.85 | 14.1046±20.2845 | 0.608 |
| PCT (ng/mL) | 1.4198±6.23042 | 1.4877±6.02849 | 0.388 |
| PT (s) | 18.7991±8.63727 | 18.7537±8.66836 | 0.849 |
| INR | 1.6505±0.92223 | 1.6515±0.91866 | 0.997 |
| Cr (mg/dL) | 0.8612±0.66269 | 0.8577±0.66077 | 0.972 |
| BUN (mmol/L) | 6.2574±12.49392 | 6.2462±12.40933 | 0.993 |
| BUN/Cr | 8.0658±14.36798 | 8.0603±14.25438 | 0.631 |
| eGFR (mL/min) | 102.3545±30.08714 | 102.6468±30.37493 | 0.99 |
| K^+^ (mmol/L) | 3.8472±0.62746 | 3.8508±0.6344 | 0.882 |
| Na^+^ (mmol/L) | 137.3887±5.17009 | 137.3973±5.17126 | 0.98 |

Note: Dataset 1 represents the unimputed dataset, and Dataset 2 represents the imputed dataset. ACLF, acute-on-chronic liver failure; ALT, alanine aminotransferase; AST, aspartate aminotransferase; AST/ALT, aspartate to alanine aminotransferase ratio; ALB, albumin; AKP, alkaline phosphatase; BUN, blood urea nitrogen; CRP, C-reactive protein; Cr, creatinine; DILI, drug-induced liver impairment; eGFR. glomerular filtration rate; HGB, hemoglobin; INR, International Normalized Ratio; K^+^, blood potassium; M (%), monocyte percentage; N (%), percent neutrophils; NLR, neutrophil-to-lymphocyte ratio; Na^+^, blood sodium; PLT, platelet; PCT, procalcitonin; PT, plasminogen time; TB, total bilirubin; WBC, white blood cell.

**Table S3.** **Comparison of Baseline Characteristics Between Training and Validation Cohorts of AoCLD Patients with SIRS.**

| **variables** | **Training Cohort**  **(n=361)** | **Validation Cohort**  **(n=154)** | ***P* value** |
| --- | --- | --- | --- |
| Demographic data |  |  |  |
| Age | 48.98 ± 11.92 | 47.26 ± 12.21 | 0.065 |
| Male | 268 (74.2%) | 117 (76%) | 0.761 |
| Aetiology | | | |
| HBV | 253 (70.1%) | 118 (76.6%) | 0.159 |
| HCV | 11 (3%) | 4 (2.6%) | 1.000 |
| HEV | 7 (1.9%) | 4 (2.6%) | 0.740 |
| Alcohol | 59 (16.3%) | 34 (22.1%) | 0.155 |
| Autoimmune | 44 (12.2%) | 13 (8.4%) | 0.277 |
| NAFLD | 14 (3.9%) | 6 (3.9%) | 1.000 |
| Schistosomiasis | 3 (0.8%) | 0 (0%) | 0.558 |
| Cryptogenic | 23 (6.4%) | 6 (3.9%) | 0.365 |
| DILI | 2 (0.6%) | 1 (0.6%) | 1.000 |
| Cirrhosis status | | | |
| Yes | 258 (71.5%) | 106 (68.8%) | 0.620 |
| AD |  |  |  |
| HE |  |  | 0.715 |
| non-HE | 332 (92.0%) | 145 (94.2%) |  |
| Grade 1-2 | 22 (6.1%) | 7 (4.5%) |  |
| Grade 3-4 | 7 (1.9%) | 2 (1.3%) |  |
| Jaundice | 183 (50.7%) | 76 (49.4%) | 0.855 |
| Ascites | 179 (49.6%) | 69 (44.8%) | 0.369 |
| GI bleeding | 49 (13.6%) | 20 (13%) | 0.970 |
| ACLF | | | |
| Yes | 73 (20.2%) | 23 (14.9%) | 0.198 |
| Blood routine | | | |
| WBC (×10^9^/L) | 5.9 ± 3.79 | 6.03 ± 3.68 | 0.459 |
| N (×10^9^/L) | 4.01 ± 3.32 | 4.06 ± 3.32 | 0.823 |
| M (×10^9^/L) | 0.5 ± 0.37 | 0.49 ± 0.28 | 0.237 |
| N (%) | 64.6 ± 13.75 | 63.91 ± 14.42 | 0.520 |
| M (%) | 8.83 ± 3.76 | 8.97 ± 3.97 | 0.804 |
| PLT (×10^9^/L) | 112.39 ± 107.16 | 115.75 ± 75.58 | 0.173 |
| HGB (g/L) | 114.1 ± 27.69 | 116.86 ± 30.08 | 0.157 |
| NLR | 4.21 ± 7.3 | 3.71 ± 3.82 | 0.303 |
| Liver function | | | |
| ALB (g/L) | 32.14 ± 6.83 | 32.72 ± 6.63 | 0.460 |
| TB (mg/dL) | 9.87 ± 10.3 | 9.91 ± 10.24 | 0.722 |
| ALT (U/L) | 379.43 ± 651.94 | 337.36 ± 506.98 | 0.713 |
| AST (U/L) | 292.86 ± 465.09 | 269.86 ± 387.27 | 0.721 |
| AST/ALT | 1.4 ± 1.07 | 1.37 ± 1.07 | 0.838 |
| AKP (U/L) | 150.16 ± 96.93 | 141.97 ± 86.54 | 0.126 |
| γ-GT (U/L) | 124.11 ± 181.54 | 117.08 ± 122.3 | 0.937 |
| Inflammation indication | | | |
| CRP (mg/L) | 13.96 ± 21.67 | 14.72 ± 17.79 | 0.077 |
| PCT (ng/mL) | 0.64 ± 1.57 | 2.06 ± 8.67 | 0.159 |
| Coagulation function | | | |
| PT (s) | 19.01 ± 9.14 | 18.67 ± 9.07 | 0.683 |
| INR | 1.68 ± 1.02 | 1.59 ± 0.6 | 0.865 |
| Kidney function | | | |
| Cr (mg/dL) | 0.87 ± 0.72 | 0.85 ± 0.5 | 0.421 |
| BUN (mmol/L) | 5.9 ± 5.29 | 7.07 ± 21.21 | 0.318 |
| BUN/Cr | 8.5 ± 16.45 | 8.71 ± 22.62 | 0.093 |
| eGFR (mL/min) | 102.5 ± 31.78 | 102.09 ± 25.42 | 0.995 |
| Electrolyte | | | |
| K^+^ (mmol/L) | 3.85 ± 0.63 | 3.86 ± 0.65 | 0.755 |
| Na^+^ (mmol/L) | 137.55 ± 5.06 | 136.99 ± 5.36 | 0.385 |
| Vital signs | | | |
| OI (mmHg) | 465.85 ± 33.54 | 467.07 ± 31.01 | 0.179 |

Note: ACLF, acute-on-chronic liver failure; ALT, alanine aminotransferase; AST, aspartate aminotransferase; AST/ALT, aspartate to alanine aminotransferase ratio; ALB, albumin; AKP, alkaline phosphatase; BUN, blood urea nitrogen; CRP, C-reactive protein; Cr, creatinine; DILI, drug-induced liver impairment; eGFR. glomerular filtration rate; GI bleeding, gastrointestinal bleeding; HBV, hepatitis B virus; HCV, hepatitis C virus; HEV, hepatitis E virus; HE, hepatic encephalopathy; HGB, hemoglobin; INR, International Normalized Ratio; K^+^, blood potassium; M, monocyte count; M (%), monocyte percentage; N, neutrophil count; N (%), percent neutrophils; NLR, neutrophil-to-lymphocyte ratio; NAFLD, nonalcoholic fatty liver disease; Na^+^, blood sodium; OI, oxygenation index; PLT, platelet; PCT, procalcitonin; PT, plasminogen time; TB, total bilirubin; WBC, white blood cell.

**Table S4. Results of the nonlinear association analysis between key variables and the risk of infection.**

| **Factor** | **Chi-Square** | **d.f.** | ***P* value** |
| --- | --- | --- | --- |
| CRP | 23.73 | 3 | <0.001 |
| Nonlinear | 18.62 | 2 | <0.001 |
| ALB | 8.76 | 3 | 0.0327 |
| Nonlinear | 1.55 | 2 | 0.4607 |
| N | 13.81 | 3 | 0.0032 |
| Nonlinear | 3.41 | 2 | 0.1815 |
| Jaundice | 4.04 | 1 | 0.0445 |
| Ascites | 2.21 | 1 | 0.1367 |
| TOTAL | 67.19 | 11 | 4.1562 |

Note: The "Nonlinear" row tests the significance of the variable's nonlinear component. *P* < 0.05: significant nonlinearity (curvilinear relationship). 0.05 ≤ *P* < 0.10: suggestive trend toward nonlinearity. *P* ≥ 0.10: no evidence of nonlinearity; treat as linear. A significant overall P-value (first row) confirms the variable is an important predictor.

**Table S5. Results of the interaction analysis between key variables.**

|  | **Estimate** | **Std. Error** | ***Z* value** | ***P* value** |
| --- | --- | --- | --- | --- |
| Intercept | -3.35072 | 1.38031 | -2.428 | 0.01520 |
| CRP | 0.08535 | 0.03955 | 2.158 | 0.03094 |
| ALB | -0.00038 | 0.03919 | -0.010 | 0.99235 |
| Jaundice 1 | 3.90257 | 1.52564 | 2.558 | 0.01053 |
| Ascites 1 | 0.76057 | 0.41118 | 1.850 | 0.06436 |
| N | 0.20521 | 0.06688 | 3.068 | 0.00215 |
| CRP:ALB | -0.00167 | 0.00124 | -1.342 | 0.17975 |
| ALB:Jaundice 1 | -0.09604 | 0.04866 | -1.974 | 0.04840 |
| CRP:Ascites1 | -0.01484 | 0.01625 | -0.913 | 0.36138 |
| CRP:N | -0.00066 | 0.00193 | -0.346 | 0.72901 |

Note: Hypothesized interaction terms are indicated by a colon (":") between variables. The "1" in "Jaundice 1" and "Ascites 1" denotes the first level of the factor variable (typically "Yes"), which is compared against the reference level ("No"). *P* < 0.05 indicates a statistically significant interaction effect.

**Table S6. Regression Coefficients and Statistical Measures for Variables in the Final Parsimonious Model.**

| **Variable** | **Coefficient (β)** | **Standard Error** | **Wald Z** | ***P* value** | **OR (95%CI)** |
| --- | --- | --- | --- | --- | --- |
| Intercept | -3.8464 | 1.4157 | -2.72 | 0.0066 | - |
| CRP (linear) | 0.2413 | 0.1685 | 1.43 | 0.1522 | 1.273(0.914-1.773) |
| CRP' (nonlinear) | -1.8602 | 2.8673 | -0.65 | 0.5165 | 0.155(0.001-38.642) |
| CRP'' (nonlinear) | 2.7093 | 4.7016 | 0.58 | 0.5644 | 15.021(0.001-225400) |
| ALB | -0.0147 | 0.0344 | -0.43 | 0.6703 | 0.985(0.921-1.054) |
| N | 0.1756 | 0.0518 | 3.39 | 0.0007 | 1.192(1.077-1.320) |
| Jaundice (Yes vs No) | 3.9188 | 1.5350 | 2.55 | 0.0107 | 50.344(2.503-1012.876) |
| Ascites (Yes vs No) | 0.4830 | 0.3202 | 1.51 | 0.1315 | 1.621(0.866-3.034) |
| ALB × Jaundice | -0.1069 | 0.0496 | -2.16 | 0.0310 | 0.899(0.815-0.991) |

Note: Probability prediction equation: P = 1 / (1 + exp(-[-3.846 + f(CRP) - 0.015×ALB + 0.176×N + 3.919×Jaundice + 0.483×Ascites - 0.107×(ALB×Jaundice)])). Where: f(CRP) = 0.241×CRP - 1.860×CRP' + 2.709×CRP'' (restricted cubic spline terms); P: predicted probability of infection; Jaundice and Ascites are categorical indicator variables (coded as 1 if present, 0 otherwise); N and ALB are continuous variables (raw values are substituted directly). ALB, albumin; CRP, C-reactive protein; CI, Confidence Interval. N, Neutrophil Count; OR, Odds Ratio.

**Table S7. Comparison between the full model and the simplified model.**

| **Model** | **AIC** | **BIC** | **AUC** | **Brier Score** |
| --- | --- | --- | --- | --- |
| Full model | 493.24 | 668.24 | 0.856 | 0.119 |
| simplified model | 361.84 | 536.84 | 0.840 | 0.127 |

Note: Lower AIC or BIC values are better. AIC, Akaike Information Criterion; AUC, Area Under the Curve; BIC, Bayesian Information Criterion.

**Figures:**


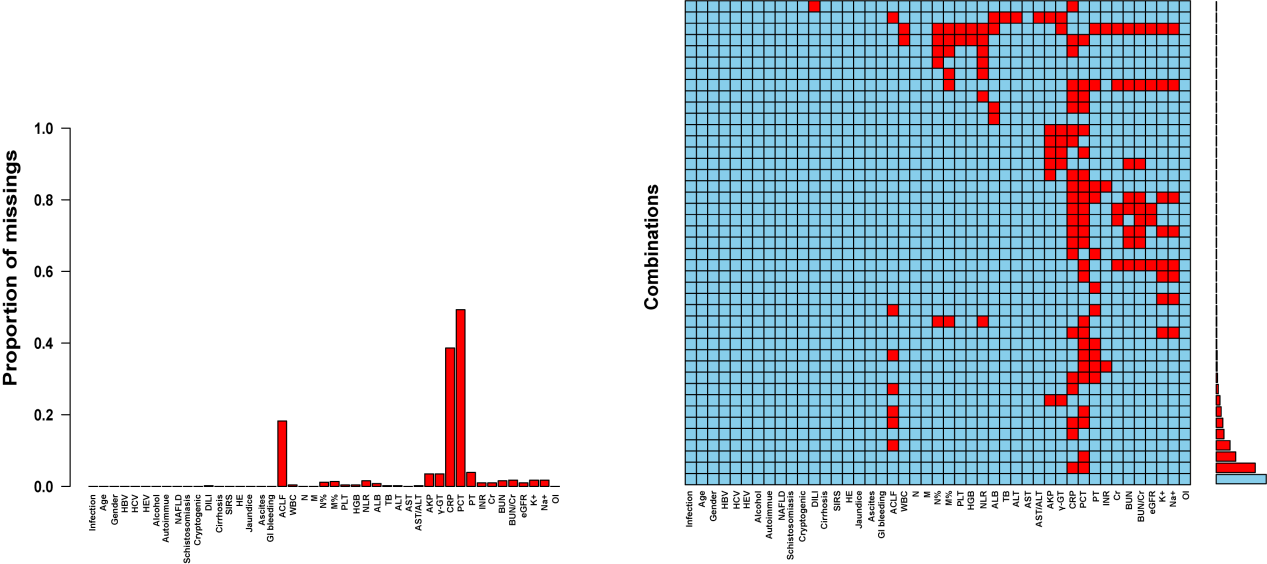


**Figure S1. Missing data display.** Bar chart of percentage of missing data (left panel). Missing data specifics display plot (right panel), red squares indicate missing data and blue squares indicate complete data. The percentage of missing data is respectively: DILI (0.1%), ACLF (18.2%), WBC (0.3%), N% (1.1%), M% (1.3%), PLT (0.3%), HGB (0.3%), NLR (1.5%), ALB (0.7%), TB (0.1%), ALT (0.1%), AST (0.1%), AST/ALT (0.1%), AKP (3.4%), GGT (3.4%), CRP (38.6%), PCT (49.3%), PT (3.8%), INR (0.9%), Cr (0.9%), BUN (1.5%), BUN/Cr (1.7%), eGFR (0.9%), K^+^ (1.7%), and Na^+^ (1.7%).


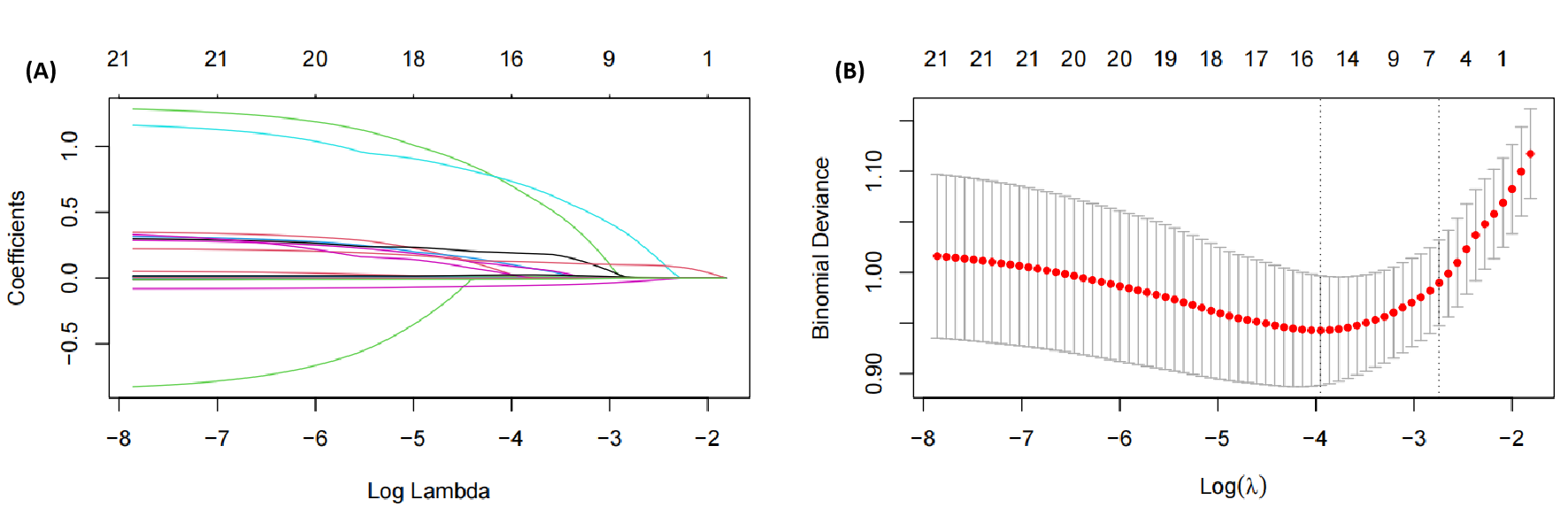


**Figure S2. LASSO Regression Analysis.** (A) LASSO Coefficient Paths: This plot shows how the regression coefficients change with log(λ). The top x-axis indicates the number of non-zero coefficients in the model, the bottom x-axis represents the standardized coefficient vector, and the y-axis displays the coefficient value of each variable. Each curve corresponds to a specific variable. (B) Cross-Validation Curve: This plot shows the mean squared error (MSE) of the LASSO regression as a function of log(λ). The top x-axis indicates the number of variables included for each λ, the bottom x-axis shows log(λ), the logarithm of the regularization parameter, and the y-axis represents the MSE, which measures the discrepancy between predicted and actual values. Each MSE value is accompanied by an error bar representing its 95% confidence interval. Two vertical dashed lines are shown: the left line corresponds to the λ that gives the minimum MSE (lambda.min), representing the model with the best prediction accuracy; the right line corresponds to the λ within one standard error of the minimum (lambda.1se), representing a more parsimonious model with fewer selected variables.


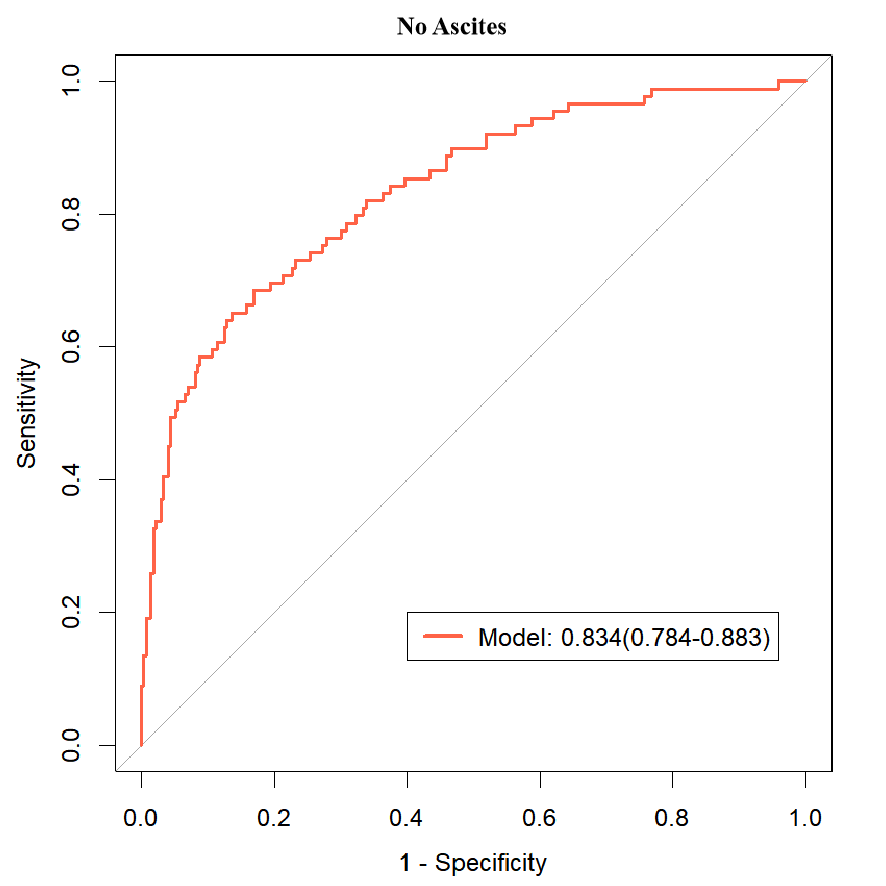


**Figure S3. ROC Curve.** An early diagnostic model for infection in AoCLD patients with SIRS was constructed using four variables: jaundice, neutrophil count, albumin, and C-reactive protein.

**
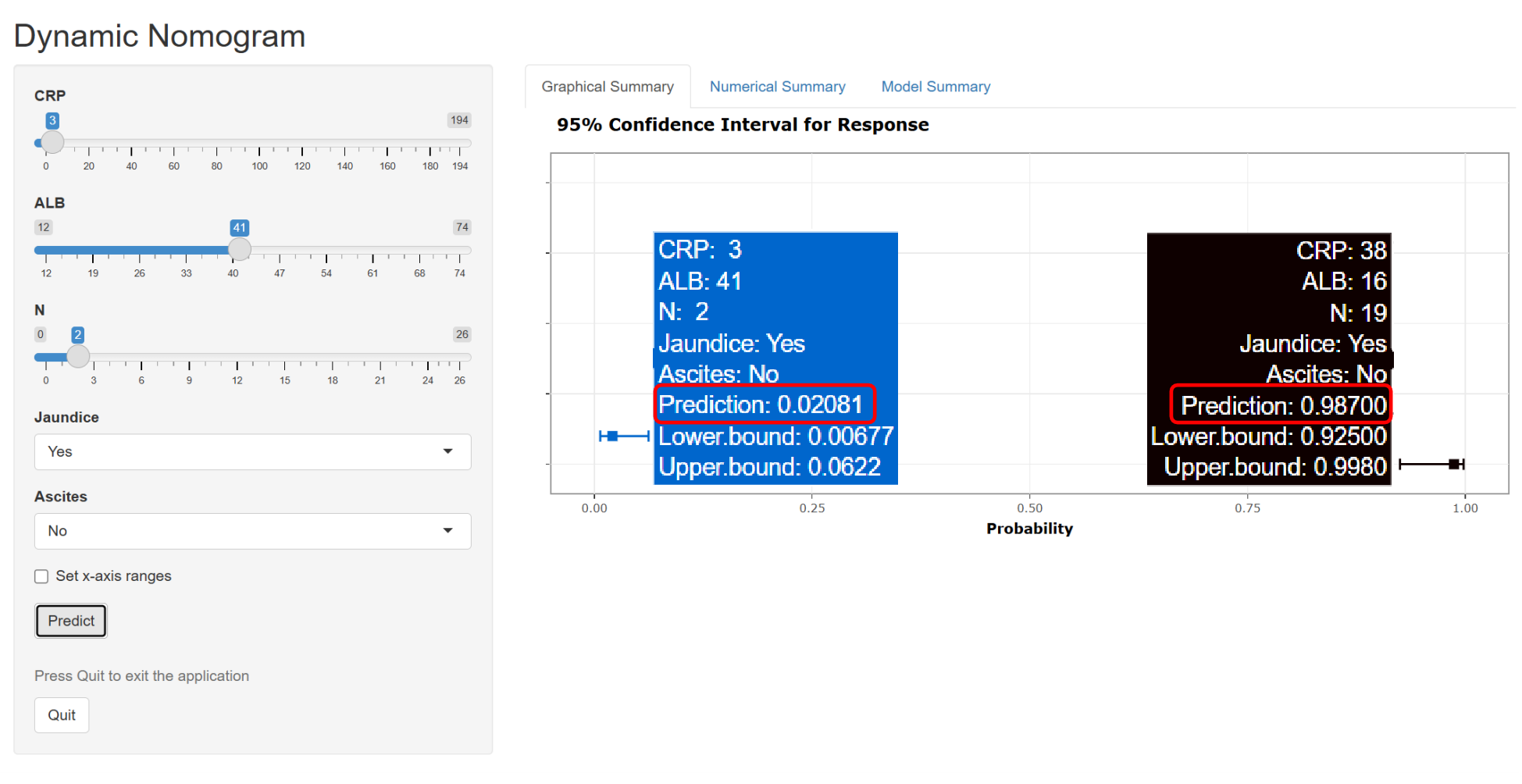
**

**Figure S4. Example outputs from the online interactive platform using clinical data of two patients.** The blue box on the left shows the test result of a non-infected patient, while the black box on the right represents the result of an infected patient.
